# Allogeneic hematopoietic stem cell transplantation for Crohn disease complicated with myelodysplastic syndrome: A case report

**DOI:** 10.1097/MD.0000000000019450

**Published:** 2020-03-06

**Authors:** Ying Zhang, Li-Li Lou, Xiao-Dong Shi, Sha-Sha Lu, Li-Xia Zhang, Xu Huang, Hui-Fan Ji, Xu Li, Xiao-Lin Guo

**Affiliations:** Department of Hepatology, The First Hospital of Jilin University, Chang chun, China.

**Keywords:** hematopoietic stem cell transplantation, inflammatory bowel disease, myelodysplastic syndrome

## Abstract

**Rationale:**

Myelodysplastic syndrome (MDS) can be complicated with Crohn disease (CD). Irritable bowel disease (IBD) associated with MDS has already been reported in the past; however, hematopoietic stem cell transplantation (HSCT) is rarely performed. Herein, we report a case of CD with MDS for HSCT.

**Patient concerns:**

A 41-year-old man was hospitalized due to abdominal pain and intermittent fever for 40 days. Two years later, he was readmitted due to abdominal pain and diarrhea with fever for 10 days.

**Diagnosis:**

Symptoms, laboratory examinations, and imaging findings of the patient were indicative of CD complicated with MDS.

**Interventions:**

An allogeneic HSCT was performed.

**Outcomes:**

He died of severe lung infection 125 days post-transplantation.

**Lessons:**

The number of cases of CD combined with MDS remains insufficient, and no consensus opinions are available to date. Hence, HSCT is a very potential treatment method. Additional experiences are needed to determine its effectiveness.

## 1 Introduction

Inflammatory bowel disease (IBD) is a chronic and recurrent intestinal disease caused by multiple factors and mediated by abnormal immunity. Its primary types are Crohn disease (CD) and ulcerative colitis. CD is a chronic granulomatous disease involving the terminal ileum and adjacent colon and is always accompanied with extraintestinal manifestations and associated immune dysfunction. Myelodysplastic syndrome (MDS) is a refractory heterogeneous disease with abnormal cytoplasm and blood cell quantity originating from hematopoietic stem cells and is characterized by hematopoietic morbidity as well as high risk for acute myeloid leukemia. Recent studies have found that MDS can be complicated with autoimmune diseases. IBD associated with MDS has already been reported previously; however, treatment with hematopoietic stem cell transplantation (HSCT) is rarely performed. Herein, we report a case of CD combined with MDS treated with HSCT.

## 2 Case presentation

**Figure 1:**
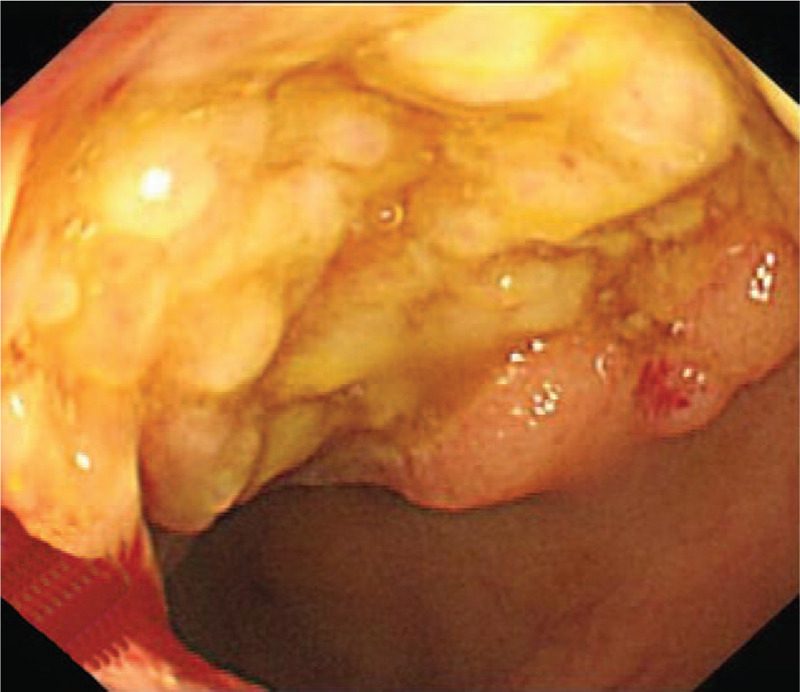
Intermittent and multiple ulcers observed at the colorectal mucosa.

**Figure 2:**
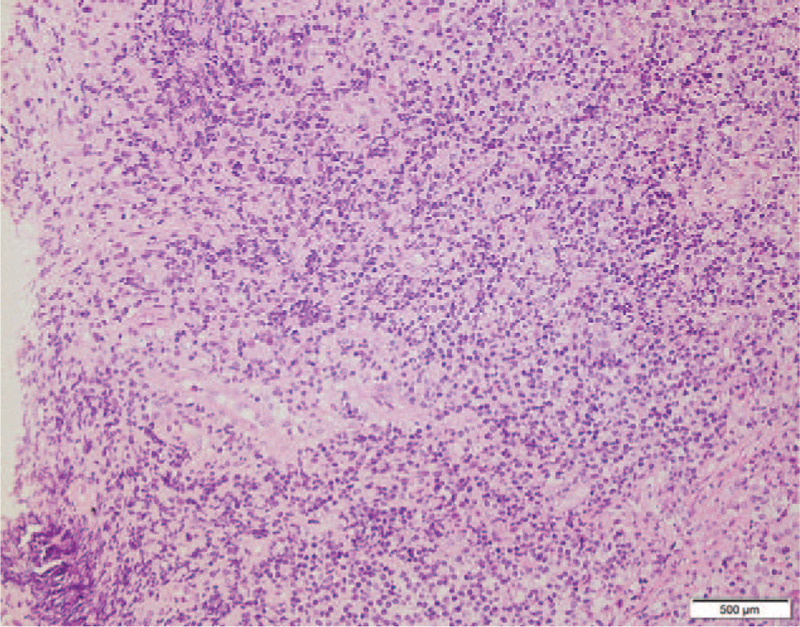
Pathological finding of the colonic biopsy specimen. Chronic mucosal inflammation, glandular hyperplasia, and necrosis are found in the ascending colon.

**Figure 3:**
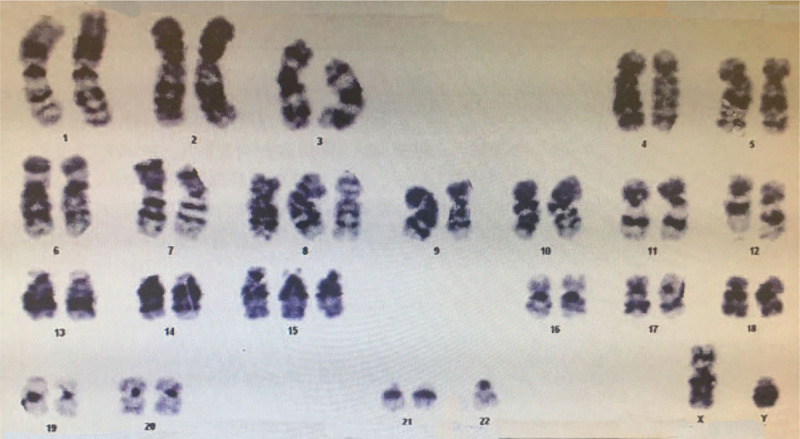
Karyotype analysis. A 47, XY, 8 karyotype was observed.

A 41-year-old man was hospitalized on December 17, 2015 due to abdominal pain and intermittent fever for 40 days. He had a 25-year history of aplastic anemia without systematic treatment and a 7-year history of follicular occlusion triad with irregular hormone therapy. Laboratory tests showed white blood cell count of 1.81 × 10^9^/L, red blood cell count of 2.39 × 10^12^/L, platelet count of 173 × 10^9^/L, hemoglobin level of 75 g/L, and erythrocyte sedimentation rate level of 44 mm/h. Bone perforation indicated increased granulocyte granules and erythroid proportion, visible morbidity accounting for 5%, and visible sub-macronucleus and phagocytosis. Additional examinations were suggested to clarify the causes of hematopenia, but he refused. Colonoscopy revealed multiple irregular ulcers at the end of the ileum, ileocecal, and ascending colon (Fig. 1). Pathology indicated chronic inflammation, edema, glandular hyperplasia, submucosal lymphoid tissue hyperplasia, and granulation tissue in the terminal mucosal mucosa. Simultaneously, chronic mucosal inflammation, glandular hyperplasia, and ascending colon necrosis were observed (Fig. 2). Based on clinical manifestations and auxiliary examination results, the patient was eventually diagnosed with CD (diagnostic criteria). Methylprednisolone sodium succinate, infliximab, mesalazine, intermittent blood transfusion, and nutritional support were administered. He was discharged after his symptoms improved but did not have regular therapy thereafter. Two years later, he was readmitted to the hospital due to abdominal pain and diarrhea with fever for 10 days. Prednisone acetate (50 mg, once a day, p.o.) and thalidomide (75 mg, once a day, p.o.) were administered. His abdominal pain and diarrhea have been alleviated. However, 1 month post-admission, dizziness and fatigue occurred. The blood routine suddenly changed. The test results displayed white blood cell count of 0.85 × 10^9^/L, absolute neutrophil count of 0.29 × 10^9^/L, red blood cell count of 1.5 × 10^12^/L, hemoglobin level of 45 g/L, and platelet count of 181 × 10^9^/L. MDS-U were diagnosed by bone marrow puncture. The chromosomal karyotype was 47 XY,+ 8 (Fig. 3). An ultra-low-dose decitabine demethylation was performed, and decitabine (10 mg, day 1–5, i.v.t.t.) was administered. The patient responded well to chemotherapy. His dizziness and fatigue have improved. However, his abdominal pain and diarrhea persisted. He still had recurrent infections, and blood transfusions were continued. One month later, his younger sister with matched human leukocyte antigen (HLA) was used as the donor for allogeneic HSCT. The pretreatment scheme was to improve BU/CY+ATG, using busulfan 4 mg/kg·d × 3, cyclophosphamide 60 mg/kg·d × 2, semustine 250 mg/(m^2^·d) × 1, and anti-human T-cell rabbit immunoglobulin 3 mg/kg·d × 2. Cyclosporine and methotrexate were administered to prevent graft-versus-host disease (GVHD). The granulocyte was implanted 20 days post-transplantation. The laboratory results showed a white blood cell count of 7.96 × 10^9^/L and absolute neutrophil value of 6.02 × 10^9^/L. The megakaryocytic series was implanted 42 days post-transplantation, and the platelets were 31 × 10^9^/L. Patient's abdominal pain and diarrhea symptoms disappeared, and his general condition improved. However, hemorrhagic cystitis, cytomegalovirus activation, and invasive pulmonary mycosis occurred. Finally, he died of severe lung infection 125 days post-transplantation.

## 3 Analysis and discussion

In recent years, reports of IBD combined with MDS have gradually increased^[1–8]^ however, no reports have investigated the incidence and prevalence of MDS. Harewood et al^[9]^ reported that the incidence of MDS in IBD was approximately 0.17% based on the investigation data. However, data were not adjusted for age, sex, or statistics and can only be used as a reference. The diagnosis of MDS in IBD is difficult, and the incidence rate may be higher. Majority of patients with IBD also had anemia and should be aware of the possibility of MDS or other blood system diseases in the presence of moderate to severe anemia or three-line abnormality that cannot be explained by common causes. The patient had a long history of anemia during the disease course; however, he did not undergo systematic treatment. During the patient's first hospitalization, his white and red blood cells decreased, and bone penetration results showed that the blood system disease was not excluded. Therefore, further examination was recommended, but the patient refused. During the second hospitalization, white and red blood cells rapidly decreased. Bone puncture and related examinations were performed again, which confirmed the MDS diagnosis. In addition, when patients with MDS develop gastrointestinal symptoms, such as abdominal pain and hematochezia, the possibility of IBD should also be considered so that the treatment prescription could be timely adjusted. The prognosis of IBD combined with MDS is poor, which mainly depends on the therapeutic effect of MDS.

At present, the mechanism of CD associated with MDS remains unclear. Based on our literature review, the following primary reasons are found. The first reason is immune abnormalities. CD is a complex autoimmune disease affected by several factors. MDS is a clonal hematopoietic progenitor/stem cell disease often associated with some autoimmune diseases. Studies have shown that patients with autoimmune diseases have a 2.1-fold increased risk of developing MDS. This occurs because autoimmune diseases share the same genetic tendency or susceptibility with MDS, thereby influencing the treatment medications for autoimmune diseases or causing direct damage to the bone marrow.^[10,11]^ The immune abnormality in CD is mainly due to the imbalance in Treg/Th17 cell differentiation. In other words, Treg secretion is insufficient, whereas Th17 secretion is excessive. Immunological abnormalities also exist in patients with MDS. Several studies found that Treg and Th17 cells are abnormal in patients with MDS, which may play an important role in the occurrence and development of MDS, but the specific mechanism remains unclear.^[12–14]^ They have the same immunological abnormalities, which influence each other to some extent. Moreover, how they interact with each other still needs further investigations. Consistent with this report, the patient had been found to have abnormal blood system results before treating CD; therefore, the patient's condition was considered to be not caused by drug factors. Instead, it is diagnosed as MDS complicated by CD. Simultaneously, the patient suffers from follicular occlusion triad, an autoimmune disease and can be complicated with CD. How these 3 diseases affect each other requires further investigation. The second reason is chromosomal abnormalities. Eng et al^[4]^ first reported that chromosomal abnormalities might be related to the concurrency of IBD and MDS. Subsequently, Nakamura et al^[5]^ further explained the relationship between the 2 chromosomal abnormalities, which are common in patients with MDS and can change the function and characteristics of bone marrow-derived antigen-presenting cells. Because the abnormal regulation of bone marrow antigen-presenting cells is involved in the occurrence of IBD, bone marrow cells with chromosomal abnormalities may cause both IBD and MDS. In addition, the decline of neutrophil activities and environmental factors in patients with MDS may also affect its occurrence. Declined neutrophil function may increase the chance of infection, an important factor influencing IBD.

Currently, the treatment for CD combined with MDS is mainly based on drug therapy due to various types of MDS and different severity of CD. A literature review found that cases of CD combined with MDS treated with HSCT were very rare. Hu et al^[2]^ reported that a 50-year-old man with CD started treatment with mesalazine and prednisone at first and then developed hematopenia. MDS (Raeb-I) was diagnosed through bone marrow examination. Then, HSCT was performed. The patient recovered well after 25 months of follow-up, and abdominal pain and diarrhea disappeared. Piccin et al^[3]^ reported a 14-year-old boy with CD who was successively treated with hydroprednisone, 6-mercaptopurine, and tumor necrosis factor alpha. Later, the anemia deteriorated; therefore, the patient had to rely on blood transfusion. Bone marrow puncture showed MDS erythrodysplasia with monosomy-7, and therefore, the patient should undergo HSCT. The patient's symptoms were relieved, and the condition was stable after the HSCT follow-up for 5 years and 8 months. Ditschkowsk et al^[7]^ reported that 11 patients with IBD with acute or chronic myelogenous leukemia or MDS (including 4 patients with UC and 7 with CD) were treated with HSCT. Except for 1 male patient with chronic myelogenous leukemia who died of pulmonary fungal infection, the remaining symptoms were relieved and patients were in good condition. After HSCT, immune memory can be eliminated, and the immune system can be reconfigured to restore the immune tolerance of chronic autoimmunity.^[15]^ Compared with the above patients, our patient has long-term irregular oral administration of hormones due to the follicular occlusion triad, and thus, increased complications during transplantation will be expected. The risk of gastrointestinal perforation, hemorrhage, GVHD, and severe infection during and after the pretreatment will also increase. Furthermore, the patient is in a state of disease progression before transplantation and needs repeated blood transfusion; therefore, the risk of transplantation is greater than that for the above patients. Although preoperative preparation was sufficient, malignant events were still not prevented. Both IBD and MDS were relieved at post-transplantation. All of the above-mentioned cases have achieved satisfactory outcomes; however, each case has its own characteristics. Pre-transplantation indicators of each patient should be rigorously evaluated in order to obtain the best treatment outcomes.

## 4 Conclusion

Although IBD combined with MDS rarely occurs, its prognosis is poor; therefore, missed diagnosis should be carefully considered. At present, opinion on treatment measures remains to be elucidated. Based on literature reports, HSCT is a potential treatment method.

## Author contributions

**Conceptualization:** Li-Li Lou.

**Data curation:** Ying Zhang.

**Methodology:** Ying Zhang, Hui-Fan Ji.

**Project administration:** Xiao-Lin Guo, Hui-Fan Ji.

**Resources:** Li-Li Lou.

**Software:** Xiao-Dong Shi.

**Supervision:** Xiao-Lin Guo, Xiao-Dong Shi, Sha-Sha Lu, Li-Xia Zhang, Xu Huang, Xu Li.

**Validation:** Xu Huang.

**Visualization:** Li-Xia Zhang, Xu Huang, Hui-Fan Ji, Xu Li.

**Writing – original draft:** Ying Zhang.

**Writing – review & editing:** Ying Zhang.
